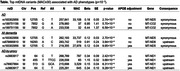# Association of mitochondrial DNA variants with Alzheimer’s disease phenotypes in 500K participants with whole genome sequencing from the UK Biobank

**DOI:** 10.1002/alz.090861

**Published:** 2025-01-03

**Authors:** Mohsen Sharifi Tabar, Biqi Cui, Chunyu Liu, Habil Zare, Claudia L Satizabal, Sudha Seshadri, Xueqiu Jian

**Affiliations:** ^1^ The University of Texas Health Science Center at San Antonio, San Antonio, TX USA; ^2^ Central South University, Changsha, Hunan China; ^3^ Boston University, Boston, MA USA

## Abstract

**Background:**

Mitochondria are organelles where energy production takes place via oxidative phosphorylation, thus mitochondrial function influences the organs with large energy consumption, such as the brain. Mitochondria contain their own circular genome (mtDNA), which encodes essential proteins/RNAs involved in oxidative phosphorylation. The maternal inheritance of mtDNA, combined with a higher risk of Alzheimer’s disease (AD) observed in females, suggest mtDNA may have a role in AD. Yet, our knowledge of the impact of mtDNA on AD remains limited, and large‐scale association studies of mtDNA variation and AD are lacking.

**Method:**

Using whole genome sequencing (WGS) data from 500K UK Biobank participants, we called mtDNA variants with MitoHPC. Three phenotypes were derived from ICD‐10 codes: AD, all dementia, and AD‐by‐proxy. Controls were free from dementia and stroke. Participants <60 years of age or non‐European white were excluded from the preliminary analysis. We assessed the association between each mtDNA variant (minor allele count ≥30) and the three phenotypes, respectively, adjusting for age, sex, assessment center, genetic principal components, and genetic relationship matrix. Additional analyses were performed with adjustment of APOE ε2/ε4 count. Significance was set at 5 × 10^−5^, or the equivalent of correcting for ∼1000 independent mtDNA haplotypes in the dataset.

**Result:**

After exclusions, up to 283,868 samples were left in our analyses, including 3742, 8459, and 53,417 with AD, all dementia, and AD‐by‐proxy, respectively. We observed suggestive associations (p<10^−3^) between mtDNA variants and AD phenotypes (**Table**), including a synonymous variant in MT‐ND5 with both AD and all dementia. Additionally, another synonymous variant in MT‐CO2 was suggestively associated with AD only. The analysis of AD‐by‐proxy identified multiple variants of interest upstream of MT‐ND1. All three genes encode essential enzymes in the electron transport chain and were previously found to be overly expressed in blood in early AD.

**Conclusion:**

Our preliminary analysis of the 500K WGS data from the UK Biobank suggested associations between multiple mtDNA variants and AD phenotypes. Robust aggregation tests suited for rare variants are underway to assess the vast majority of the mtDNA variants (rarer) identified by WGS. Inclusion of diverse samples is warranted to reveal ancestry‐specific effect of mtDNA variation on AD.